# Clinical Outcomes and Patient Satisfaction Following WALANT Hand and Wrist Surgery in a Regional Island Hospital: A Retrospective Observational Study From the Maldives

**DOI:** 10.7759/cureus.117802

**Published:** 2026-10-12

**Authors:** Nay Aung Zin, Niaz N Mahmud, Phyo Thant, Nan Aung, Phyu S Thwin, Brang Jar

**Affiliations:** 1 Orthopaedics and Traumatology, Kulhudhuffushi Regional Hospital, Kulhudhuffushi, MDV; 2 Emergency, Kulhudhuffushi Regional Hospital, Kulhudhuffushi, MDV; 3 Orthopaedics and Traumatology, University of Medicine 1, Yangon, Yangon, MMR

**Keywords:** ambulatory surgery, hand surgery, maldives, patient satisfaction, quickdash, regional hospital, walant

## Abstract

Background

Wide Awake Local Anaesthesia No Tourniquet (WALANT) is increasingly used for hand and wrist surgery because it permits procedures to be performed without sedation, general anaesthesia, or pneumatic tourniquet application. These characteristics may be particularly advantageous in regional healthcare systems with limited anaesthesia and operating theatre resources. However, evidence regarding WALANT outcomes in regional island healthcare settings such as the Maldives remains limited.

Objective

To evaluate clinical outcomes, postoperative functional status, patient satisfaction, recovery characteristics, and complications following hand and wrist procedures performed using the WALANT technique at a regional hospital in the Maldives.

Methods

We retrospectively analysed routinely collected operative records, completed questionnaires, and follow-up data of 62 adults who underwent WALANT hand or wrist surgery at Kulhudhuffushi Regional Hospital between June 2024 and May 2025. A commercially premixed solution of 2% lignocaine with adrenaline 1:80,000 was used without sodium bicarbonate. Outcomes included perioperative visual analogue scale pain scores, postoperative QuickDASH scores, patient experience, recovery, and complications. Complete data for the reported outcomes were available for all 62 included patients.

Results

The mean age was 44.5 ± 12.8 years, and 38 patients (61.3%) were female. Carpal tunnel syndrome was the most common diagnosis (24/62, 38.7%). Mean VAS pain scores were 3.10 ± 1.30 during local anaesthetic injection and 1.40 ± 0.79 intraoperatively. Fifty-five patients (88.7%) described the intraoperative experience as comfortable or very comfortable. Same-day discharge was achieved in 56 patients (90.3%), 50 (80.6%) returned to daily activities within one week, and 54 (87.1%) returned to work within four weeks. The mean postoperative QuickDASH score was 28.50 ± 12.93. Fifty-four patients (87.1%) were satisfied or very satisfied with their WALANT experience, and 57 (91.9%) indicated that they would undergo WALANT again if required. Five patients (8.1%) had recorded postoperative complications, comprising two wound infections (3.2%), two cases of transient numbness (3.2%), and one haematoma (1.6%). One patient undergoing a trauma-related fracture procedure required reoperation because of poor fracture alignment. No major anaesthetic complication or digital ischaemia was recorded. No conversion to general anaesthesia was required.

Conclusions

In this single-centre series, WALANT was feasible for selected hand and wrist procedures in a regional island hospital and was associated with low perioperative pain, high patient satisfaction, and frequent same-day discharge. Postoperative complications were recorded in five patients (8.1%), with no major anaesthetic complications or digital ischaemia observed. These findings support further evaluation of WALANT as an ambulatory surgical approach in comparable regional and resource-constrained healthcare settings. Prospective multicentre comparative studies incorporating baseline and longitudinal functional assessment are warranted.

## Introduction

Wide Awake Local Anaesthesia No Tourniquet (WALANT) has emerged as an increasingly adopted anaesthetic approach for hand and wrist surgery. The technique uses local anaesthesia combined with epinephrine to provide analgesia and haemostasis while avoiding sedation, general anaesthesia, and pneumatic tourniquet application [1,2]. WALANT has been used across a range of elective and trauma-related hand procedures, allowing appropriately selected operations to be performed while the patient remains awake and cooperative [2,3].

Conventionally, hand surgery may be performed under general anaesthesia, regional anaesthesia, or local anaesthesia with pneumatic tourniquet application. Although these techniques provide effective operative conditions, tourniquet use can cause discomfort, while sedation or general anaesthesia requires additional perioperative resources and recovery. WALANT provides an alternative that avoids tourniquet-related discomfort and routine sedation [1,4]. In selected procedures, particularly flexor tendon surgery, the awake patient can actively move the digits during surgery, allowing assessment of tendon gliding, repair integrity, and potential impingement before completion of the procedure [5,6].

The use of epinephrine in digital and hand surgery was historically limited by concerns regarding digital ischaemia and tissue necrosis. Subsequent evidence, however, has challenged the traditional prohibition against appropriately administered epinephrine in the fingers [7,8]. Clinical series have also demonstrated the use of lidocaine with epinephrine for hand surgery without digital circulatory compromise [3]. More recent systematic evidence has further supported a low incidence of ischaemic complications when WALANT is used in appropriately selected patients [9].

Patient comfort and acceptance are important considerations because patients remain awake throughout WALANT procedures. Previous studies have reported favourable patient experiences with wide-awake hand surgery. Gunasagaran et al. reported greater perceived comfort with WALANT compared with local anaesthesia using a tourniquet [4]. Davison et al. demonstrated favourable patient acceptance of wide-awake carpal tunnel surgery [10], while Moscato et al. reported high patient satisfaction following carpal tunnel release using WALANT [11]. A recent systematic review and meta-analysis also demonstrated high levels of patient satisfaction among adults undergoing elective hand surgery using WALANT [12].

In addition to patient experience, WALANT may facilitate ambulatory surgical pathways by reducing dependence on sedation and post-anaesthetic recovery. Cost analysis of carpal tunnel release has demonstrated potential economic advantages when procedures are performed wide awake rather than under sedation [13]. A systematic review and meta-analysis comparing WALANT with local or intravenous regional anaesthesia using a tourniquet also reported favourable postoperative pain outcomes without evidence of increased complications [14].

These potential advantages may be particularly relevant in regional and resource-constrained healthcare settings. The Maldives comprises geographically dispersed island communities, where regional hospitals may have more limited access to specialist anaesthesia services, operating theatre capacity, and perioperative resources than major tertiary centres. In such settings, WALANT may offer practical advantages by allowing appropriately selected hand and wrist procedures to be performed without routine sedation, general anaesthesia, or pneumatic tourniquet use. At Kulhudhuffushi Regional Hospital, WALANT has been used for a range of elective and trauma-related procedures, including carpal tunnel release, trigger finger release, De Quervain release, ganglion excision, tendon repair, and selected trauma procedures. However, evidence describing clinical outcomes and patient experience following WALANT surgery in regional island healthcare settings remains limited. Therefore, this study aimed to evaluate perioperative pain, postoperative functional status, recovery characteristics, patient satisfaction, and complications following WALANT hand and wrist surgery in a regional island hospital in the Maldives.

## Materials and methods

Study design and setting

This single-centre retrospective observational study was conducted in the Department of Orthopaedics and Trauma at Kulhudhuffushi Regional Hospital, Maldives. We analysed existing operative records, questionnaires, and follow-up data of patients who underwent hand or wrist surgery using wide-awake local anaesthesia no tourniquet (WALANT) between June 1, 2024, and May 31, 2025. These data were collected during routine care before the analysis began.

Study population

Adults aged 18 years or older who underwent a WALANT hand or wrist procedure during the study period and had sufficient clinical and follow-up records were eligible. Patients with incomplete records or insufficient follow-up information were excluded. Patients with a known allergy to lignocaine or adrenaline, severe peripheral vascular disease, or compromised digital circulation were not considered suitable for WALANT.

All 62 eligible patients were included. No formal sample-size calculation was performed. Complete records for the reported outcomes were available for all 62 included patients, and none was lost to follow-up. Anxiety status was recorded for 42 patients.

Surgical procedures

The procedures included carpal tunnel release, trigger finger release, De Quervain release, ganglion excision, tendon repair, and selected K-wire fixation or other trauma-related hand procedures.

WALANT technique

A commercially premixed preparation of 2% lignocaine with adrenaline 1:80,000 (Lignox 2% A) was used for local infiltration. Sodium bicarbonate was not added. The volume was selected according to the operative site and the patient’s body weight, with a planned maximum lignocaine dose of 7 mg/kg or 500 mg, whichever was lower. The solution was infiltrated slowly into the operative field, with aspiration to reduce the risk of intravascular injection. Approximately 20-30 minutes were allowed after infiltration before incision. No pneumatic tourniquet or routine sedation was used.

The operative hand was prepared with 4% chlorhexidine solution and draped before incision. Sterile gloves and instruments were used. Patients remained awake during surgery. Active movement was assessed when clinically relevant, particularly during tendon procedures, to evaluate tendon gliding, repair integrity, and finger movement.

Blood pressure, pulse rate, respiratory rate, and oxygen saturation were monitored during the procedure. Patients were assessed for clinical features of local anaesthetic systemic toxicity during surgery and postoperative observation. Digital circulation was also checked (Figure 1).

**Figure 1:**
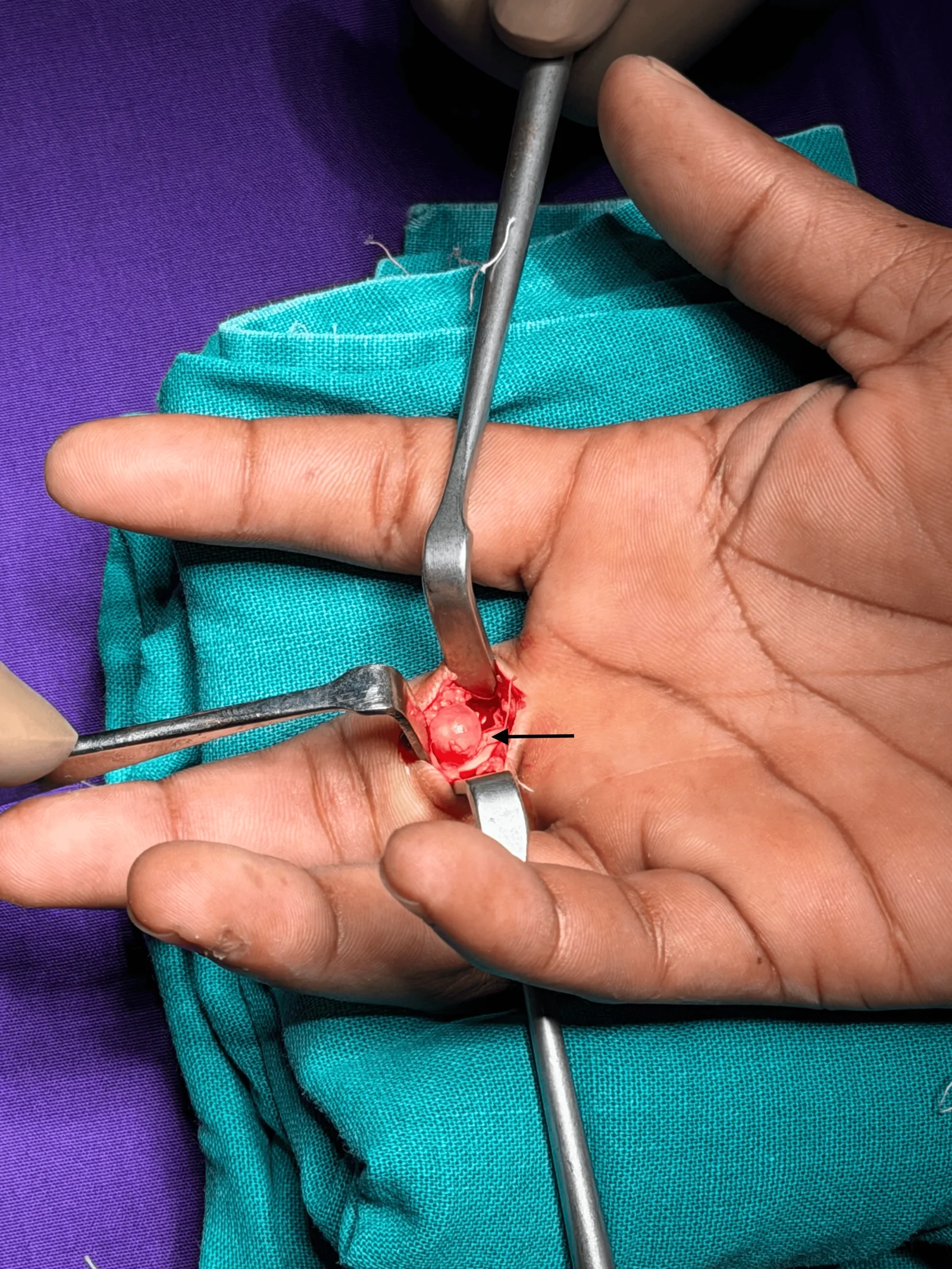
Intraoperative ganglion excision performed using the WALANT technique. Representative intraoperative photograph demonstrating a ganglion cyst (arrow) during excision under Wide Awake Local Anaesthesia No Tourniquet (WALANT). The procedure was performed using local anaesthesia with epinephrine without pneumatic tourniquet application or routine sedation. The hand was prepared and draped before incision. The photograph shows the operative exposure; it does not depict the skin-preparation step.

Data collection and outcome measures

Data were extracted from existing operative records, medical records, follow-up assessments, and completed patient questionnaires. Variables included age, sex, hand dominance, affected side, diagnosis, procedure performed, requirement for additional local anaesthesia, and complications.

Pain intensity was recorded using a 0-10 visual analogue scale (VAS), where 0 indicated no pain and 10 indicated the worst imaginable pain [15]. Scores recorded during local anaesthetic injection, intraoperatively, immediately postoperatively, at 24 hours, and at one week were analysed.

Postoperative upper-limb functional status was assessed at approximately four weeks using the 11-item Quick Disabilities of the Arm, Shoulder and Hand (QuickDASH) questionnaire [16]. Scores range from 0 to 100, with lower scores indicating less disability. As preoperative QuickDASH scores were unavailable, the results describe postoperative functional status and cannot establish improvement from baseline.

Patient experience and recovery were assessed using a six-item questionnaire developed locally for routine postoperative follow-up. It was not adapted from a published instrument and had not undergone formal validation. The items addressed intraoperative comfort, overall satisfaction, willingness to undergo WALANT again, same-day discharge, return to usual daily activities within one week, and return to work within four weeks. Each response was analysed separately as a categorical outcome; no total questionnaire score was calculated. The original English-language questionnaire and response options are provided in the Appendix.

Patients were assessed at approximately 24 hours and one week, with subsequent follow-up according to the procedure and clinical need. The mean follow-up duration was 6.2 ± 1.8 weeks (range: 4 to 12 weeks).

Statistical analysis

Data were analysed using IBM SPSS Statistics for Windows, Version 26.0 (IBM Corp., Armonk, NY, USA). Continuous variables were summarised as means and standard deviations, and categorical variables as frequencies and percentages. Procedure-specific outcomes were described without formal between-procedure hypothesis testing because the procedure groups were small, unequal in size, and clinically heterogeneous.

Ethical considerations

The project was registered with the Quality Improvement Department of Kulhudhuffushi Regional Hospital (reference KRH/QID/AUD/2026/002). Although registered through the hospital’s audit/quality improvement process, this manuscript reports a retrospective observational analysis of routinely collected outcomes. It was not designed as an audit against a predefined standard, and no baseline audit or re-audit was performed. According to the institutional determination for this project, formal ethics committee review and individual consent for analysis of anonymised routinely collected data were not required. Data were de-identified before analysis.

## Results

Patient characteristics

A total of 62 patients who underwent hand and wrist procedures using the Wide Awake Local Anaesthesia No Tourniquet (WALANT) technique were included in the analysis. Complete follow-up and questionnaire records were available for all 62 included patients at the reported assessment points. None was lost to follow-up. Anxiety status was recorded for 42 patients. The mean age was 44.5 ± 12.8 years, with a range of 19-68 years. The largest age group was 31-45 years (24/62, 38.7%), followed by 46-60 years (20/62, 32.3%).

Thirty-eight patients (61.3%) were female and 24 (38.7%) were male. Most patients were right-hand dominant (58/62, 93.5%). The right side was affected in 36 patients (58.1%), the left side in 22 (35.5%), and bilateral involvement was recorded in four (6.5%). Baseline demographic and clinical characteristics are summarized in Table 1.

**Table 1:** Demographic characteristics of patients undergoing hand and wrist surgery using the WALANT technique (n=62). Data are presented as number and percentage unless otherwise indicated. The mean patient age was 44.5 ± 12.8 years (range: 19-68 years). WALANT: Wide Awake Local Anaesthesia No Tourniquet.

Characteristic	n (%)
Age group	
18-30 years	10 (16.1)
31-45 years	24 (38.7)
46-60 years	20 (32.3)
>60 years	8 (12.9)
Sex	
Male	24 (38.7)
Female	38 (61.3)
Hand dominance	
Right	58 (93.5)
Left	4 (6.5)
Side affected	
Right	36 (58.1)
Left	22 (35.5)
Bilateral	4 (6.5)

Case mix and procedures

Carpal tunnel syndrome was the most frequent diagnosis, accounting for 24 cases (38.7%), followed by trigger finger in 14 (22.6%), De Quervain disease in eight (12.9%), ganglion cyst in six (9.7%), tendon injury in five (8.1%), and finger trauma in five (8.1%).

The corresponding procedures were carpal tunnel release, trigger finger release, De Quervain release, ganglion excision, tendon repair, and K-wire fixation or other trauma-related procedures. The distribution of procedures is illustrated in Figure 2.

**Figure 2:**
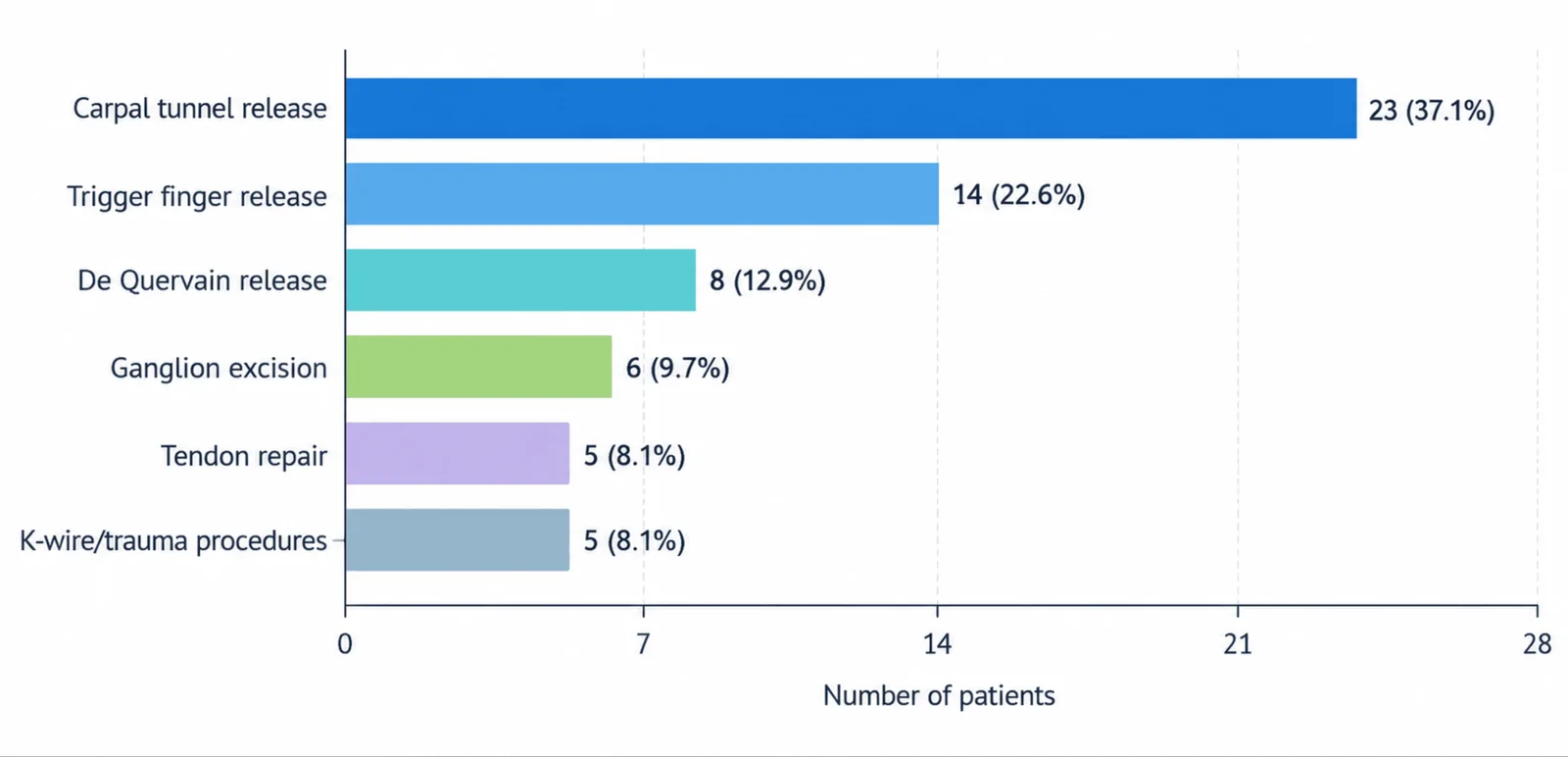
Distribution of hand and wrist procedures performed using the WALANT technique (n=62). The horizontal bars represent the number of patients undergoing each procedure. Carpal tunnel release was the most frequently performed procedure (24/62, 38.7%), followed by trigger finger release (14/62, 22.6%). WALANT: Wide Awake Local Anaesthesia No Tourniquet.

Intraoperative outcomes

The mean VAS pain score during local anaesthetic injection was 3.10 ± 1.30, while the mean intraoperative VAS score was 1.40 ± 0.79. Most patients reported a favourable intraoperative experience: 25 (40.3%) described the procedure as very comfortable and 30 (48.4%) as comfortable, giving a combined comfortable or very comfortable rate of 88.7%. Five patients (8.1%) reported a neutral experience, while two (3.2%) described the procedure as uncomfortable. Anxiety status was documented in 42 patients (67.7%). Among those with documented anxiety status, 30 (71.4%) had mild anxiety, 10 (23.8%) had moderate anxiety, and two (4.8%) had severe anxiety. Anxiety status was not recorded in 20 patients (32.3%). Additional local anaesthetic supplementation was required in four patients (6.5%). Minimal intraoperative bleeding was documented in 57 patients (91.9%), and no conversion to general anaesthesia was recorded (Table 2).

**Table 2:** Intraoperative pain, patient experience, anxiety, anaesthetic supplementation, and operative characteristics during WALANT surgery (n=62). Continuous variables are presented as mean ± standard deviation (SD), and categorical variables as number/denominator (percentage). Visual Analogue Scale (VAS) pain scores were measured on a 0-10 scale, with higher scores indicating greater pain. Intraoperative comfort and operative outcomes are reported using the total study population (n=62) as the denominator. Anxiety status was documented in 42 patients; therefore, percentages for mild, moderate, and severe anxiety were calculated using the 42 patients with recorded anxiety data as the denominator. Anxiety status was not recorded in 20 patients (32.3%), and these missing data were not interpreted as absence of anxiety. WALANT: Wide Awake Local Anaesthesia No Tourniquet

Outcome	Result
Pain assessment	
VAS during local anaesthetic injection, mean ± SD	3.10 ± 1.30
Intraoperative VAS, mean ± SD	1.40 ± 0.79
Intraoperative comfort	
Very comfortable	25/62 (40.3%)
Comfortable	30/62 (48.4%)
Neutral	5/62 (8.1%)
Uncomfortable	2/62 (3.2%)
Anxiety assessment	
Anxiety status documented	42/62 (67.7%)
Mild anxiety	30/42 (71.4%)
Moderate anxiety	10/42 (23.8%)
Severe anxiety	2/42 (4.8%)
Anxiety status not recorded	20/62 (32.3%)
Anaesthetic and operative characteristics	
Additional local anaesthetic required	4/62 (6.5%)
Minimal intraoperative bleeding	57/62 (91.9%)
Conversion to general anaesthesia	0/62 (0%)

Perioperative and postoperative pain

Pain scores remained low throughout the assessed perioperative and postoperative periods. The mean VAS score was 3.10 ± 1.30 during local anaesthetic injection and 1.40 ± 0.79 intraoperatively. The mean immediate postoperative VAS score was 2.60 ± 1.04, decreasing to 2.10 ± 0.93 at 24 hours and 1.20 ± 0.65 at one week. The pattern of mean pain scores across the assessment points is shown in Figure 3.

**Figure 3:**
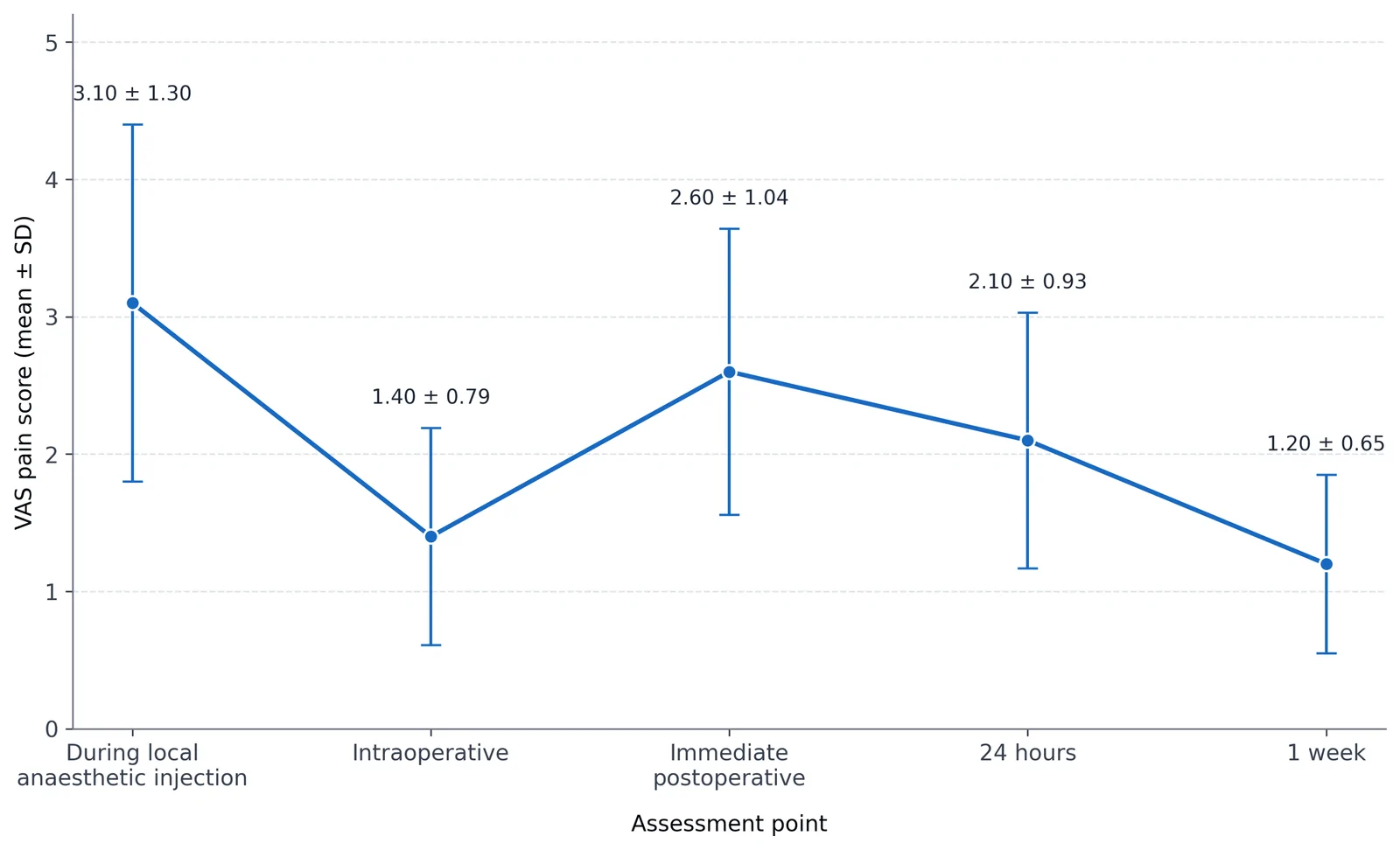
Mean Visual Analogue Scale pain scores during and following WALANT surgery. Mean VAS pain scores were assessed during local anaesthetic injection, intraoperatively, immediately postoperatively, at 24 hours, and at one week. Values are presented as mean ± standard deviation. The figure is descriptive and does not represent a formal test of change over time. VAS: Visual Analogue Scale; WALANT: Wide Awake Local Anaesthesia No Tourniquet

Functional recovery and patient-reported outcomes

The mean postoperative QuickDASH score was 28.50 ± 12.93, with scores ranging from 10 to 61. As preoperative QuickDASH measurements were not available, this value represents postoperative functional status rather than the magnitude of functional improvement attributable to surgery.

Same-day discharge was achieved in 56 patients (90.3%). Fifty patients (80.6%) returned to daily activities within one week, while 54 (87.1%) returned to work within four weeks.

Patient-reported experience was favourable. Fifty-four patients (87.1%) were satisfied or very satisfied with their WALANT experience, and 57 (91.9%) stated that they would undergo WALANT again if another suitable procedure were required. In addition, 55 patients (88.7%) described the intraoperative experience as comfortable or very comfortable. These key patient-centred and recovery outcomes are summarized visually in Figure 4.

**Figure 4:**
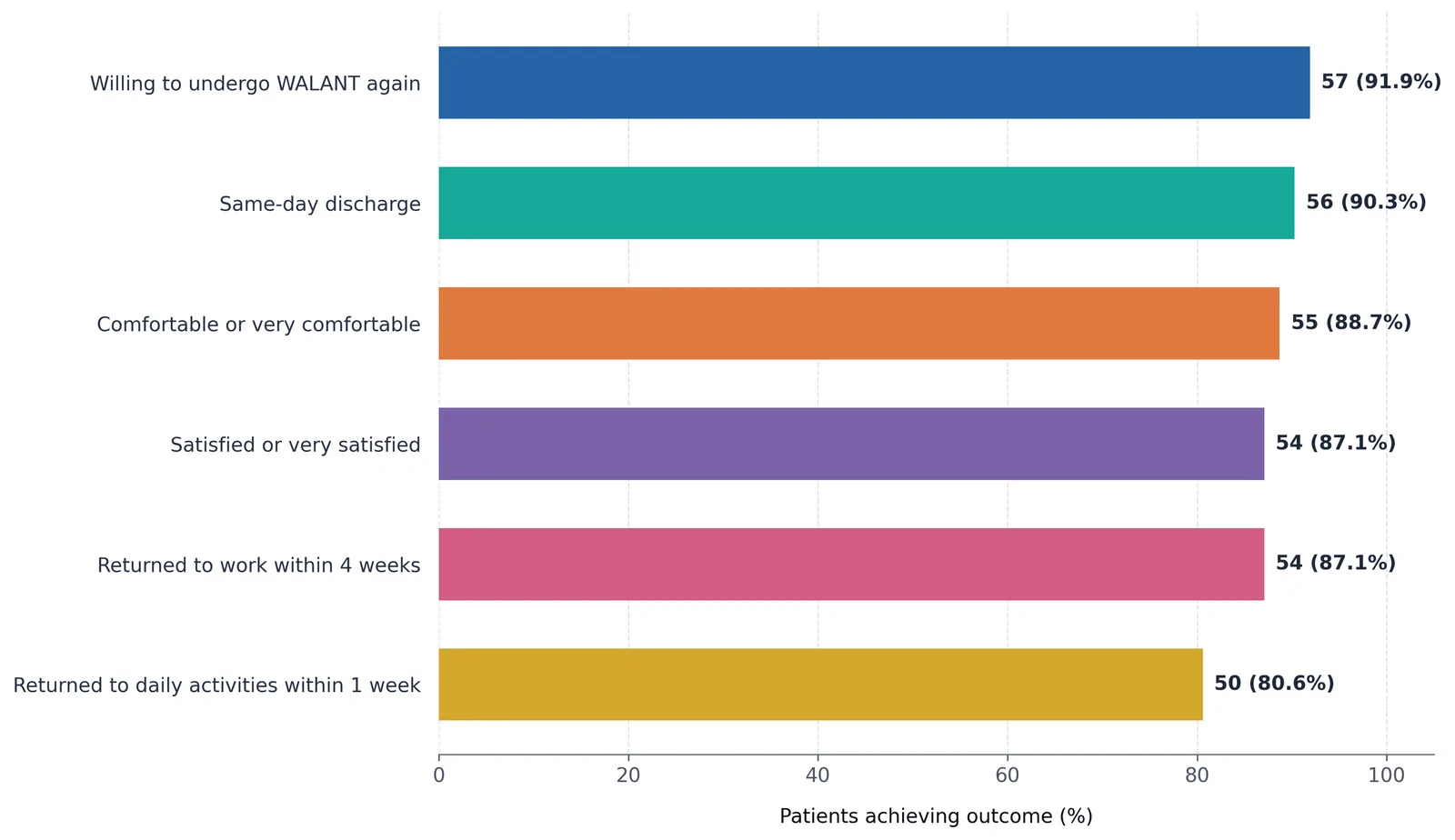
Key recovery and patient-reported outcomes following WALANT hand and wrist surgery. Bars represent the number and percentage of the total study population (n=62) achieving each specified outcome. Fifty-seven patients (91.9%) were willing to undergo WALANT again, 56 (90.3%) achieved same-day discharge, 55 (88.7%) described the procedure as comfortable or very comfortable, 54 (87.1%) were satisfied or very satisfied, 54 (87.1%) returned to work within four weeks, and 50 (80.6%) returned to daily activities within one week. WALANT: Wide Awake Local Anaesthesia No Tourniquet.

Procedure-specific outcomes

Procedure-specific outcomes were examined descriptively. Mean intraoperative VAS scores were low across all procedure categories, ranging from 0.90 ± 0.48 for tendon repair to 1.72 ± 0.72 for ganglion excision.

The overall postoperative QuickDASH score was 28.50 ± 12.93. Procedure-specific mean QuickDASH scores ranged from 20.60 ± 5.03 following K-wire fixation/trauma procedures to 30.83 ± 12.11 following ganglion excision.

Satisfaction was high across most procedure groups. All patients undergoing De Quervain release and tendon repair were satisfied or very satisfied, while three of five patients (60.0%) undergoing K-wire fixation/trauma procedures reported satisfaction. Because several procedure groups contained small numbers of patients and represented different underlying conditions, these comparisons were considered descriptive and no formal between-procedure hypothesis testing was performed (Table 3).

**Table 3:** Procedure-specific intraoperative pain, postoperative functional status, and patient satisfaction following WALANT surgery. Values are presented descriptively because of the small and unequal numbers of patients within individual procedure categories. QuickDASH was assessed postoperatively; no preoperative QuickDASH measurement was available. Therefore, QuickDASH values represent postoperative functional status and should not be interpreted as change from baseline. VAS: Visual Analogue Scale; QuickDASH: Quick Disabilities of the Arm, Shoulder and Hand; SD: standard deviation; WALANT: Wide Awake Local Anaesthesia No Tourniquet.

Procedure	n	Intraoperative VAS, mean ± SD	Postoperative QuickDASH, mean ± SD	Satisfied/very satisfied, n (%)
Carpal tunnel release	24	1.28 ± 0.83	29.42 ± 14.77	21 (87.5)
Trigger finger release	14	1.67 ± 0.76	29.79 ± 14.62	12 (85.7)
De Quervain release	8	1.27 ± 0.81	27.38 ± 11.50	8 (100)
Ganglion excision	6	1.72 ± 0.72	30.83 ± 12.11	5 (83.3)
Tendon repair	5	0.90 ± 0.48	27.40 ± 7.80	5 (100)
K-wire fixation/trauma procedure	5	1.52 ± 0.93	20.60 ± 5.03	3 (60.0)

Complications and reoperation

Five patients (8.1%) had a postoperative complication recorded in the master dataset. Wound infection occurred in two patients (3.2%), transient numbness in two (3.2%), and haematoma formation in one (1.6%).

One patient (1.6%) undergoing a trauma-related fracture procedure required reoperation because of poor fracture alignment. The indication for reoperation was therefore related to fracture alignment rather than an anaesthetic complication attributable to WALANT.

No tendon rupture or repair failure, major anaesthetic complication, or digital ischaemia was recorded in the study population. Complications and adverse outcomes are summarized in Table 4.

**Table 4:** Postoperative complications, reoperation, and adverse outcomes following hand and wrist surgery using WALANT. Complications are presented as number and percentage of the total study population (n=62). One patient required reoperation because of poor fracture alignment following a trauma-related fracture procedure; this was considered a procedure-related event rather than a WALANT-related anaesthetic complication. The reoperation category should not be added to the five recorded postoperative complications to calculate an overall complication rate because these variables are recorded separately in the master dataset. WALANT: Wide Awake Local Anaesthesia No Tourniquet.

Outcome	n (%)
Patients with a recorded postoperative complication	5 (8.1)
Wound infection	2 (3.2)
Transient numbness	2 (3.2)
Haematoma formation	1 (1.6)
Reoperation due to poor fracture alignment	1 (1.6)
Tendon rupture/repair failure	0 (0)
Major anaesthetic complication	0 (0)
Digital ischaemia	0 (0)

## Discussion

The present study evaluated clinical outcomes and patient experience following WALANT hand and wrist surgery in a regional island hospital in the Maldives. The principal findings were low perioperative pain scores, favourable intraoperative comfort, frequent same-day discharge, early return to daily activities and work, and high patient satisfaction. The mean intraoperative VAS score was 1.40 ± 0.79, 88.7% of patients described the procedure as comfortable or very comfortable, and 90.3% achieved same-day discharge. Overall, 87.1% were satisfied or very satisfied, and 91.9% indicated that they would undergo WALANT again if required. No major anaesthetic complication or digital ischaemia was recorded. These findings suggest that WALANT was feasible and well accepted for appropriately selected hand and wrist procedures in this regional hospital setting.

Pain and intraoperative experience

Adequate pain control is particularly important during WALANT because patients remain awake throughout the procedure. In the present study, the mean VAS pain score during local anaesthetic injection was 3.10 ± 1.30 and decreased to 1.40 ± 0.79 intraoperatively. Mean postoperative VAS scores were 2.60 immediately after surgery, 2.10 at 24 hours, and 1.20 at one week.

These findings are consistent with previous evidence supporting satisfactory perioperative analgesia with WALANT. Gunasagaran et al. reported greater perceived comfort among patients undergoing minor hand surgery using WALANT compared with local anaesthesia with tourniquet use [4]. A systematic review and meta-analysis by Evangelista et al. also reported lower postoperative pain with WALANT compared with local or intravenous regional anaesthesia with tourniquet use [14]. Avoidance of pneumatic tourniquet inflation may contribute to patient comfort by eliminating tourniquet-associated discomfort while local infiltration provides operative analgesia.

In the present study, 55 patients (88.7%) described their intraoperative experience as comfortable or very comfortable. Only four (6.5%) required additional local anaesthetic supplementation, and no conversion to general anaesthesia was recorded. These findings support the practical acceptability of the technique within the study population. Nevertheless, appropriate patient selection, preoperative counselling, careful local anaesthetic infiltration, assessment of anxiety, and readiness to manage potential adverse events remain important components of WALANT practice.

Patient satisfaction and acceptance

Patient acceptance was high, with 54 patients (87.1%) reporting that they were satisfied or very satisfied and 57 (91.9%) stating that they would undergo WALANT again if another suitable procedure were required.

These findings are consistent with previous reports of favourable patient experience following wide-awake hand surgery. In a prospective cohort study of carpal tunnel release, Davison et al. reported that 93% of patients undergoing wide-awake surgery would choose the same anaesthetic approach again [10]. Moscato et al. also reported favourable patient-reported outcomes following WALANT carpal tunnel release [11], while the systematic review and meta-analysis by Siu et al. demonstrated high levels of patient satisfaction across elective WALANT hand surgery studies [12].

Willingness to undergo WALANT again is a clinically meaningful outcome because it reflects the overall patient experience rather than pain alone. Avoidance of sedation, tourniquet discomfort, and prolonged post-anaesthetic recovery may contribute to patient acceptance. However, individual preferences differ, and some patients may experience anxiety while remaining awake during surgery. Appropriate counselling and shared decision-making therefore remain important.

Recovery and ambulatory surgery

Recovery characteristics were favourable in this cohort. Same-day discharge was achieved in 56 patients (90.3%), 50 (80.6%) returned to daily activities within one week, and 54 (87.1%) returned to work within four weeks.

Avoidance of routine sedation or general anaesthesia may reduce the requirement for post-anaesthetic monitoring and facilitate ambulatory surgical pathways. In a prospective study, Davison et al. reported shorter hospital time among patients undergoing wide-awake carpal tunnel release compared with those receiving intravenous sedation [10]. Alter et al. also demonstrated potential cost advantages for carpal tunnel release performed wide awake rather than under sedation [13].

Nevertheless, the present study did not include a conventional-anaesthesia comparator or formal economic analysis. Therefore, the observed same-day discharge and recovery outcomes cannot be attributed solely to WALANT, and conclusions regarding cost-effectiveness or reductions in healthcare resource utilization cannot be made from the present data.

Postoperative functional status

The mean postoperative QuickDASH score was 28.50 ± 12.93. Procedure-specific mean scores varied among the diagnostic and surgical groups. These findings require cautious interpretation because QuickDASH was assessed only postoperatively and no preoperative baseline measurement was available.

Consequently, the QuickDASH findings represent postoperative functional status rather than functional improvement attributable to WALANT or surgery. Functional recovery after hand surgery is influenced by multiple factors, including the underlying diagnosis, severity of injury, type of procedure, rehabilitation, and duration of follow-up. Anaesthetic technique represents only one component of the overall treatment pathway.

This issue is particularly relevant because the study included both elective soft-tissue conditions and trauma-related procedures with different expected recovery trajectories. Procedure-specific QuickDASH values were therefore considered descriptive. Future prospective studies should incorporate preoperative QuickDASH assessment and standardized postoperative measurements at multiple time points to evaluate longitudinal functional recovery.

Complications and safety considerations

Five patients (8.1%) had recorded postoperative complications, comprising two wound infections, two cases of transient numbness, and one haematoma. These postoperative events should be distinguished from complications directly attributable to the WALANT anaesthetic technique, such as local anaesthetic systemic toxicity, epinephrine-associated digital ischaemia, or inadequate anaesthesia. The recorded wound infections, transient numbness, and haematoma may have been related to the underlying pathology or surgical intervention, although their precise causes cannot be established retrospectively. One patient undergoing a trauma-related fracture procedure required reoperation because of poor fracture alignment. This was considered a procedure-related complication rather than a WALANT-related anaesthetic complication. No major anaesthetic complication or digital ischaemia was recorded in 62 patients. Nevertheless, the small sample size and retrospective design prevent definitive conclusions regarding the comparative safety of WALANT.

Concerns regarding epinephrine-associated digital ischaemia have historically represented a barrier to wide-awake hand surgery. Thomson et al. critically reviewed the evidence surrounding elective epinephrine use in the finger [7], while Denkler examined the historical evidence concerning digital necrosis associated with epinephrine [8]. Subsequent clinical experience has supported the use of appropriately administered epinephrine-containing local anaesthesia. Sardenberg et al. reported 488 hand procedures performed using local anaesthesia with epinephrine without tourniquet, sedation, or an anaesthesiologist [3].

Rougereau et al. subsequently evaluated ischaemic complications associated with WALANT in a systematic review [9]. In addition, Abdullah et al. reported a large series of 1,073 WALANT finger and hand procedures [17]. Lawand et al. more recently conducted a systematic review and meta-analysis examining complications and adverse effects associated with WALANT upper-limb surgery [18].

The complications observed in the present study should not automatically be considered WALANT-related adverse events. Wound infection, haematoma, transient numbness, and fracture malalignment may be associated with the underlying injury or surgical procedure rather than the anaesthetic technique. Conversely, the absence of major anaesthetic complications or digital ischaemia among only 62 patients cannot establish definitive safety or exclude uncommon adverse events. The present findings therefore support observed feasibility rather than proving comparative safety.

Relevance to a regional island healthcare setting

A distinctive feature of this study is its regional island healthcare setting. The Maldives comprises geographically dispersed island communities, creating logistical challenges for specialist surgical care outside major tertiary centres. Within such an environment, an anaesthetic approach that permits selected hand and wrist procedures to be performed without routine sedation, general anaesthesia, or pneumatic tourniquet use may have practical value.

The present series included common elective procedures such as carpal tunnel release, trigger finger release, De Quervain release, and ganglion excision, together with tendon repairs and selected trauma procedures. Previous large clinical series have similarly demonstrated the applicability of WALANT across a range of hand procedures [3,17].

Potential resource advantages are also relevant in this setting. Wide-awake surgery may reduce dependence on sedation and post-anaesthetic recovery pathways, while previous economic studies have demonstrated potential cost advantages for selected procedures [13]. However, the present study did not directly compare theatre utilization, anaesthesia staffing, costs, waiting times, or length of stay with conventional anaesthetic pathways. Therefore, system-level or economic benefits within the Maldivian healthcare system require further evaluation.

WALANT should not be considered a replacement for conventional anaesthesia in all patients or procedures. Procedure complexity, vascular status, patient preference and anxiety, surgeon experience, availability of appropriate monitoring and rescue medication, and institutional protocols remain important considerations. WALANT is better considered an additional anaesthetic strategy for appropriately selected patients within a suitably governed surgical service.

Strengths and limitations

An important strength of this study is that it provides real-world data on WALANT implementation in a regional island hospital, a healthcare environment that is underrepresented in the existing literature. The cohort included several commonly performed elective and trauma-related hand and wrist procedures. In addition to perioperative pain and complications, patient-centred outcomes including comfort, satisfaction, willingness to undergo WALANT again, return to daily activities, return to work, and postoperative functional status were evaluated.

Several limitations should be acknowledged. First, this was a retrospective, single-centre observational study and was therefore susceptible to selection bias, incomplete documentation, and unmeasured confounding. Second, the overall sample size was relatively small, particularly within individual procedure categories, limiting subgroup comparisons and the ability to identify uncommon complications. Third, there was no comparator group undergoing general anaesthesia, regional anaesthesia, or local anaesthesia with tourniquet use; consequently, comparative effectiveness and safety cannot be established.

Fourth, QuickDASH was measured only postoperatively without a preoperative baseline, preventing assessment of change in functional status. Fifth, the cohort contained heterogeneous diagnoses and procedures with different expected recovery trajectories. Sixth, follow-up was relatively short and may not have identified late complications, recurrence, or longer-term functional outcomes. Patient-reported measures may also have been affected by recall and response bias, and some variables, including anxiety, were incompletely documented in the retrospective dataset. In addition, patient experience was assessed using a locally developed questionnaire that had not undergone formal validation, which may limit the reliability and external comparability of these outcomes.

Finally, no formal health-economic analysis was undertaken. Although WALANT may offer logistical advantages in a regional setting, the present study cannot establish cost-effectiveness. Prospective multicentre comparative studies incorporating standardized patient selection, baseline and longitudinal patient-reported outcome measures, longer follow-up, comparator anaesthetic techniques, and formal health-economic evaluation would provide stronger evidence regarding the role of WALANT within the Maldivian healthcare system and comparable regional settings.

## Conclusions

This single-centre retrospective observational study of 62 patients suggests that WALANT is a feasible and well-accepted anaesthetic approach for selected hand and wrist procedures in a regional island hospital in the Maldives. Perioperative pain scores were low, 88.7% of patients described the intraoperative experience as comfortable or very comfortable, 90.3% achieved same-day discharge, 87.1% were satisfied or very satisfied, and 91.9% indicated that they would undergo WALANT again if required.

Postoperative complications were recorded in five patients (8.1%), with no major anaesthetic complications or digital ischaemia observed. One patient required reoperation for poor fracture alignment following a trauma-related procedure, which was considered procedure-related rather than directly attributable to WALANT.

Although these findings suggest that WALANT may be a potentially useful ambulatory anaesthetic approach in regional and resource-constrained healthcare settings, this interpretation remains hypothesis-generating. The relatively small sample size, retrospective single-centre design, absence of a comparator group, short follow-up, and lack of preoperative QuickDASH assessment preclude definitive conclusions regarding comparative safety, effectiveness, functional improvement, or resource advantages. Larger prospective multicentre comparative studies incorporating longitudinal patient-reported outcomes and health-economic evaluation are warranted.

## Appendices

Appendix: Locally developed postoperative questionnaire

**Table 5:** Original English-language patient-experience and recovery questionnaire used during routine follow-up. The six items were analysed separately. Comfort and satisfaction used five response categories; willingness to undergo WALANT again, same-day discharge, and return to usual activities used yes/no responses. Return to work used yes/no/not applicable responses. No total questionnaire score was calculated. This locally developed questionnaire was not formally validated. WALANT: Wide Awake Local Anaesthesia No Tourniquet.

Item	Question	Response options
1	How comfortable were you during the operation?	Very comfortable; comfortable; neutral; uncomfortable; very uncomfortable
2	How satisfied were you with your overall WALANT surgical experience?	Very satisfied; satisfied; neutral; dissatisfied; very dissatisfied
3	Would you undergo WALANT again if another suitable operation were required?	Yes; no
4	Were you discharged from the hospital on the same day as the operation?	Yes; no
5	Did you return to your usual daily activities within one week?	Yes; no
6	Did you return to work within four weeks?	Yes; no; not applicable
